# Depletion of Bone Marrow-Derived Fibrocytes Attenuates TAA-Induced Liver Fibrosis in Mice

**DOI:** 10.3390/cells8101210

**Published:** 2019-10-07

**Authors:** Felix Hempel, Martin Roderfeld, Rajkumar Savai, Akylbek Sydykov, Karuna Irungbam, Ralph Schermuly, Robert Voswinckel, Kernt Köhler, Yury Churin, Ladislau Kiss, Jens Bier, Jörn Pons-Kühnemann, Elke Roeb

**Affiliations:** 1 Department of Gastroenterology, Justus Liebig University, D-35392 Giessen, Germany; felix.hempel@med.jlug.de (F.H.); martin.roderfeld@innere.med.uni-giessen.de (M.R.); kukuirungbam@gmail.com (K.I.); yury.churin@innere.med.uni-giessen.de (Y.C.); 2 Max Planck Institute for Heart and Lung Research, Member of the German Center for Lung Research (DZL), Member of the Cardio-Pulmonary Institute (CPI), D-61231 Bad Nauheim, Germany; rajkumar.savai@mpi-bn.mpg.de; 3 Department of Internal Medicine, Cardio-Pulmonary Institute (CPI), Universities of Giessen and Marburg Lung Center (UGMLC), Member of the German Center for Lung Research (DZL), Justus Liebig University, D-35392 Giessen, Germany; akylbek.sydykov@innere.med.uni-giessen.de (A.S.); ladislau.kiss@innere.med.uni-giessen.de (L.K.); jens.bier@innere.med.uni-giessen.de (J.B.); 4 Department of Internal Medicine, Bürgerhospital, D-61169 Friedberg, Germany; robert.voswinckel@gz-wetterau.de; 5 Department of Internal Medicine, Hochwaldkrankenhaus, D-61231 Bad Nauheim, Germany; 6 Institute of Veterinary Pathology, Justus Liebig University, D-35392 Giessen, Germany; kernt.koehler@vetmed.uni-giessen.de; 7 Institute of Medical Informatics, Justus Liebig University, D-35392 Giessen, Germany; joern.pons@informatik.med.uni-giessen.de

**Keywords:** fibrocytes, liver fibrosis, bone marrow, myofibroblasts, thioacetamide (TAA), HSV-TK

## Abstract

Bone marrow-derived fibrocytes (FC) represent a unique cell type, sharing features of both mesenchymal and hematopoietic cells. FC were shown to specifically infiltrate the injured liver and participate in fibrogenesis. Moreover, FC exert a variety of paracrine functions, thus possibly influencing the disease progression. However, the overall contribution of FC to liver fibrosis remains unclear. We aimed to study the effect of a specific FC depletion, utilizing a herpes simplex virus thymidine kinase (HSV-TK)/Valganciclovir suicide gene strategy. Fibrosis was induced by oral thioacetamide (TAA) administration in C57BL/6J mice. Hepatic hydroxyproline content was assessed for the primary readout. The HSV-TK model enabled the specific depletion of fibrocytes. Hepatic hydroxyproline content was significantly reduced as a result of the fibrocyte ablation (−7.8%; 95% CI: 0.7–14.8%; *p* = 0.033), denoting a reduced deposition of fibrillar collagens. Lower serum alanine transaminase levels (−20.9%; 95% CI: 0.4–36.9%; *p* = 0.049) indicate a mitigation of liver-specific cellular damage. A detailed mode of action, however, remains yet to be identified. The present study demonstrates a relevant functional contribution of fibrocytes to chronic toxic liver fibrosis, contradicting recent reports. Our results emphasize the need to thoroughly study the biology of fibrocytes in order to understand their importance for hepatic fibrogenesis.

## 1. Introduction

Liver fibrosis is denoted by the excess deposition of extracellular matrix (ECM) components in response to chronic liver injury, such as viral hepatitis, cholestatic disorders, alcoholic liver disease or non-alcoholic fatty liver disease (NAFLD). With perpetuated injury, liver fibrosis might progress to cirrhosis and facilitate hepatocellular carcinoma (HCC) formation [1]. Fibrosis accounts for severe morbidity and mortality and has been shown to determine the outcome of patients with NAFLD [2].

Bone marrow-derived fibrocytes (FC) represent a unique cell type, sharing features of both hematopoietic and mesenchymal cells. While their secretion of collagens and other ECM components resembles fibroblasts, they express various leucocyte markers (e.g., CD34, CD45, CD11b, Ly6C, and F4/80) [3,4] and are hence commonly identified by the simultaneous expression of CD45 and collagen I [5]. Fibrocytes comprise ∼0.5% of peripheral blood leucocytes and rapidly enter the site of injury in physiological and pathological wound healing processes [3,6]. Since they had been explicitly described by Bucala et al. in 1994 [3], FC have been shown to participate in fibrotic diseases of the lung [7], kidney [8], heart [9], and colon [10,11]. Moreover, they are implicated in the pathogenesis of asthma [12], inflammatory bowel disease [10,13], and ocular disorders [14].

In recent years, the mechanisms of hepatic fibrogenesis have been studied extensively. Activated hepatic stellate cells (HSCs) and, to a lesser extent, portal fibroblasts were identified as the main source of contractile, α-SMA^+^ myofibroblasts in the liver, which, being absent under healthy conditions, drive scar tissue formation during hepatic fibrogenesis [15,16,17]. HSCs are therefore commonly considered the key to understanding and treating liver fibrosis [15,18,19]. However, there is compelling evidence that FC, in fact, contribute to liver fibrosis. Fate-tracing studies demonstrated that fibrocytes specifically infiltrate the liver upon injury [20] and participate in fibrogenesis by the secretion of ECM components [21,22]. Furthermore, FC constitute a potential source of myofibroblasts. Although the transdifferentiation into myofibroblasts has been shown both in vitro [7,23] and in vivo [12], its relevance remains controversial.

Besides their direct contribution to fibrogenesis, FC exert a variety of paracrine functions (reviewed in references [24,25]), thus possibly influencing liver fibrosis. FC, on the one hand, express the fibrogenic mediators TGF-β and PDGF [24,26], which are essential for the activation and proliferation of myofibroblasts [1,27]. FC can acquire an inflammatory phenotype, characterized by the production of cytokines (e.g., TNF-α, IL-1β, and CCL2,-3,-4) and eicosanoids [26,28], and their capability of antigen presentation [29]. On the other hand, FC are able to promote the degradation of ECM components via the secretion of matrix metalloproteinases (MMPs) [30,31], regulate angiogenesis [32,33], and exert antimicrobial defense mechanisms [34].

Given the complex interplay of the aforementioned factors in the pathogenesis of liver fibrosis, the overall contribution of FC remains highly speculative. We therefore seek to characterize the role of FC on experimental liver fibrosis in vivo by specific depletion of these cells. Utilizing the well-established herpes simplex virus thymidine kinase (HSV-TK)/Valganciclovir (VCV) model, driven by a collagen I promotor, we depleted collagen I-expressing cells of bone marrow (BM) origin [35,36,37,38] in C57BL/6J mice. All collagen-producing cells herein co-express the HSV-TK, making them susceptible to killing by Valganciclovir. BM of such mice was transplanted into non-transgenic mice in order to limit the effect to cells of BM origin. Introducing the HSV-TK via bone marrow transplantation circumnavigates the issue of an unstable expression of the widely used markers CD34 and, as shown recently, CD45 in fibrocytes [5]. Additionally, the collagen-promotor driven expression of HSV-TK enables the killing of fibrocytes in various stages of their development or differentiation. Although a CD14^+^ cell population, located in the bone marrow, is assumed to be the origin of fibrocytes [23], the exact differentiation pathways remain poorly understood. Thus, the results and possible side effects of an approach that interferes with alleged monocyte precursors in order to deplete fibrocytes, as reported recently [39], seem hardly predictable to us.

While different animal models of murine liver fibrosis have been described [40], with CCl_4_-induced liver injury certainly being the most popular, we chose to induce fibrosis with thioacetamide (TAA). TAA, administered via drinking water, causes a chronic-toxic, more slowly progressing fibrosis with only moderately increased serum transaminase levels, thus closely mimicking alcoholic liver fibrosis in humans [41,42]. The aim of the present study was two-fold: To determine whether the depletion of fibrocytes (1) ameliorates fibrosis, as indicated by decreased hepatic hydroxyproline content, and (2) attenuates liver cell damage, denoted by reduced serum alanine transaminase levels.

## 2. Materials and Methods

### 2.1. Animal Experiments

The present study was performed with permission of the State of Hesse, Regierungspraesidium Giessen, according to section 8 of the German Law for the protection of animals and conforms to the NIH guide for the care and use of laboratory animals. All experiments were approved by the committee on the ethics of animal experiments of the Regierungspraesidium Giessen, Germany (permit number: V54-19c 20 15c GI20/10 Nr. G21_2016 and JLU Nr. 532_M). Col-HSV-TK mice were generated at the Max Planck Institute for Heart and Lung Research animal facility (Bad Nauheim, Germany), as described previously [38]. In brief, purified Col1-HSV-TK-IRES-EGFP plasmids (Supplementary Figure S1a) were microinjected into the pronucleus of a fertilized ovum obtained from super-ovulated female mice. Subsequently, groups of injected embryos were re-implanted into the oviducts of pseudo-pregnant female mice. The litters were bred with wild-type C57BL/6J mice. Female, positively genotyped mice were paired again with wild-type mice to create a heterozygous Col-HSV-TK colony. After genotyping, offspring were used for the experiments. All mice were housed in a pathogen-free environment under a constant 12-hour light-dark cycle at 22 °C temperature and 50% humidity. The mice were fed standard chow (ALTROMIN, Lage, Germany) and water ad libitum.

**Figure 1:**
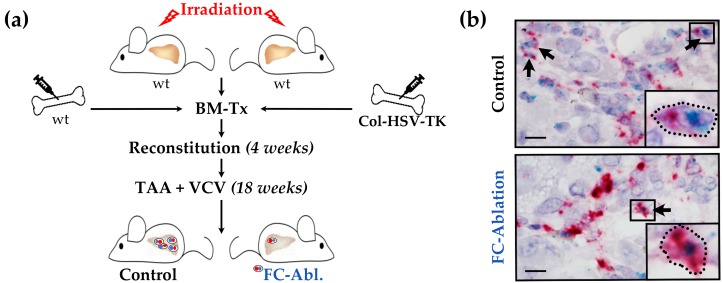
Suicide gene strategy enabled fibrocyte depletion. (**a**) Schematic representation of the animal experiments including lethal irradiation, bone marrow transplantation (BM-Tx), and treatment with valganciclovir (VCV) and thioacetamide (TAA). While TAA induces hepatic fibrosis, VCV is metabolized into toxic compounds by all cells expressing herpes simplex virus thymidine kinase (HSV-TK). (**b**) Successful depletion was confirmed by RNA in situ hybridization. Fibrocytes (FC, black arrows) were identified by the simultaneous expression of *Col1a1* (red) and *Ptprc* (CD45, blue) transcripts. The details in boxes were enlarged in individual panels in the lower right part of the micrograph. Note that individual and possibly *Col1a1/Ptprc* co-expressing cells were detected in the fibrocyte-ablated group. Magnification 1000×, bars 10 µm.

5 × 10^6^ bone marrow (BM) cells were transplanted from male Col-HSV-TK (FC-Ablation) or C57BL/6J wild-type mice (Control) into 12 weeks old lethally irradiated (11 Gy, ^60^Co) female C57BL/6J mice via tail vein injection (*n* = 16 for each group). For FC depletion during fibrogenesis after 4 weeks of reconstitution 300 mg/l TAA (Sigma-Aldrich, Munich, Germany) and 8.3 mg/l VCV (Roche, Basel, Switzerland) were administered orally via drinking water for 18 weeks. Mice were sacrificed at the age of 34 weeks. A summary of the animal experiment is depicted schematically in Figure 1a.

For FC ablation during regeneration, TAA but not VCV was given until the age of 34 weeks. Thereafter, the TAA-administration was stopped and VCV was added during a 4-week regeneration period. Untreated female C57BL/6J mice, sacrificed at the age of 34 and 38 weeks, served as supercontrols (SC). Liver samples were shock frosted and stored at −80 °C or preserved for histology as indicated below. Serum samples were stored at −80 °C until analysis of alanine aminotransferases (ALT) by routine clinical chemistry on a Reflotron Plus Analyzer (Roche, Mannheim, Germany).

### 2.2. RNA in Situ Hybridization Assay

Liver samples were fixed in 1% paraformaldehyde for 12 h, embedded in paraffin and cut into 5 µm sections. RNAscope^®^ 2.5 HD Duplex RNA in situ hybridization assay (Advanced Cell Diagnostics, Newark, CA, USA) was performed according to the manufacturer’s instructions, applying standard pretreatment conditions [43]. The ethanol incubation following target retrieval was performed for ten minutes and the ninth amplification step was extended to one hour. Specific probes were used for the detection of type I collagen- (*Mm-Col1a1*, #319379) and CD45- (*Mm-Ptprc*, #318651) gene expression. Positive- and negative-controls were carried out using probes specific to murine housekeeping-genes (*Mm-Ppib/Mm-Polr2a*, #321651) and a bacterial gene (*dapB*, #320751).

### 2.3. Histology and Immunohistochemistry

Paraffin-embedded liver samples were cut into 3–6 µm sections and routine hematoxylin/eosin and Masson’s trichrome staining were performed. For Sirius Red/Fast Green staining, sections were deparaffinized, hydrated, incubated in a staining solution consisting of 0.1% Sirius Red (Polysciences, Inc., Warrington, PA, USA) and 0.1% Fast Green (Roth, Karlsruhe, Germany) in saturated picric acid (Chroma, Münster, Germany) for one hour and differentiated in 1% acetic acid for 45 seconds.

To perform immunohistochemical stainings, peroxidase activity was blocked with 3% hydrogen peroxide. Afterwards, sections were boiled for 10 minutes either in citrate buffer (pH 6.0, Collagen I), Tris-EDTA buffer (pH 9.0, CD45) or no target retrieval was performed (α-SMA). Sections were then blocked with 10% BSA (PAA, Pasching, Austria) and 2.5% normal horse serum (Vector Laboratories, Inc., Burlingame, CA, USA) and incubated with specific antibodies (Rabbit anti Collagen I polyclonal antibody, ab34710, Abcam, Cambridge, UK; Rabbit anti CD45 polyclonal antibody, 20103-1-AP, Proteintech, Rosemont, IL, USA; Mouse anti α-SMA monoclonal antibody, 61001, Progen, Heidelberg, Germany), diluted 1:200 in 10% BSA in PBS. Secondary antibodies coupled with horseradish peroxidase (MP-7401/MP-7452) or alkaline phosphatase (MP-5401) and corresponding substrates (SK-4100) were used for detection (all purchased from Vector Laboratories, Inc., Burlingame, CA, USA). Unspecific isotype IgGs were used to control the specificity of the secondary antibodies. Sections were counterstained with hematoxylin to visualize nuclei.

All sections were eventually dehydrated and mounted with Pertex^®^ (Medite, Burgdorf, Germany). Photographs were taken using a Leica DMRB microscope (Leica, Wetzlar, Germany) equipped with a Canon EOS 600D with Canon EOS Utility 2 software, version 2.14 (Canon, Tokyo, Japan).

### 2.4. Pathological Staging and Grading

Hepatic staging and grading were performed by a trained pathologist (K.K.) in a blinded fashion. Hematoxylin/eosin and Masson’s trichrome-stained sections were evaluated, utilizing the scoring system suggested by Ishak et al. [44].

### 2.5. Morphometric Analysis

As many liver lobules as possible were photographed in two Sirius Red/Fast Green-stained sections of each mouse (magnification 200×, blinded for groups). Images suitable for analysis were identified following predefined exclusions criteria and the red stained area was quantified using the color threshold tool in ImageJ, version 1.51 [45]. To quantify the stained area in CD45 immunohistochemical sections, as many non-overlapping high-power fields as possible were photographed using a Biozero BZ-8000 microscope (magnification 200×, Keyence, Osaka, Japan). Suitable images were converted to 8-bit grey scale and the threshold tool in ImageJ was used to quantify the stained area.

### 2.6. Hydroxyproline Assay

Total hepatic hydroxyproline content was quantified as described previously [41].

### 2.7. Quantitative Real-Time PCR

RNA extraction from full liver lysates and elimination of genomic DNA was performed using the RNeasy Mini- (QIAGEN, Hilden, Germany) and TURBO DNAfree-Kit (Thermo Fisher Scientific, Waltham, MA, USA), each following the manufacturer’s instructions. RNA integrity and purity were assessed by gel electrophoresis and spectrophotometry, equal amounts of RNA were then subjected to cDNA synthesis, using the iScript cDNA Synthesis-Kit (Bio-Rad, Hercules, CA, USA). qPCR was carried out, including one of the primer pairs listed in Supplementary Table S2 and SYBR-Green/ROX dye. *Hprt* was validated and used as a reference gene. Statistical tests and computation of confidence intervals were performed on ΔC_T_-values, calculated as

ΔC_T_ = C_T_(reference gene) − C_T_(gene of interest). (1)

Fold-changes were calculated as

fold-change = 2^ΔC_T_(FC − Abl.) − ΔC_T_(Ctrl)^. (2)

### 2.8. Gene Expression Array

84 fibrosis-related genes were analyzed using the RT^2^ Profiler™ PCR Array Mouse Fibrosis (PAMM-120ZC, QIAGEN, Hilden, Germany) according to the manufacturer’s instructions. RNA was prepared as described above and cDNA synthesis was performed with RT^2^ First Strand Kit (QIAGEN, Hilden, Germany) on pooled samples (fibrocyte-ablated and control group, *n* = 15 per group). Data analysis was conducted utilizing the QIAGEN data analysis web portal.

### 2.9. Western Blot Analysis

Western blot experiments were performed as described previously [46] using 1:1.000 diluted antibodies against α-SMA (Mouse anti α-SMA monoclonal antibody, 61001, Progen, Heidelberg, Germany), Bax (Rabbit anti Bax polyclonal antibody, #2772), and Bcl-2 (Rabbit anti Bcl-2 polyclonal antibody, #2876). Rabbit anti α-Tubulin polyclonal antibodies (#2144, all purchased from Cell Signaling Technology, Inc., Danvers, MA, USA) or Mouse anti ß-Actin monoclonal antibodies (sc-47778, Santa Cruz Biotechnology, Inc., Dallas, TX, USA) were used for loading controls.

### 2.10. Multiplex ELISA

Mouse Magnetic Luminex Assay (LXSAMSM, R&D Systems, Minneapolis, MN, USA) was performed according to the manufacturer’s protocol to quantify hepatic protein levels of fibrosis- and inflammation-relevant factors. Luminex^®^ 200 flow-cytometer and xPONENT^®^ software, version 3.1 (Luminex Corp., Austin, TX, USA), were used for measurements and data analysis.

### 2.11. Proteome Profiling

Mouse XL Cytokine Array Kit (ARY028, R&D Systems, Minneapolis, MN, USA) was used to test pooled samples (*n* ≥ 15 per group) of native liver lysates according to the manufacturer’s instructions. Regions of interest (ROIs) were defined on high resolution scans of the membranes in ImageJ and the mean grey values (OD) were retrieved. After subtraction of the lowest OD (OD_background_), every pair of ROIs was assigned a relative OD-value relative to the pair of ROIs with the highest OD (OD_reference_) using the equation

(3) $${\mathrm{Relative}\;\mathrm{OD}}_{\mathrm{ROI}}\;[\%]\;=\;\frac{{\mathrm{OD}}_{\mathrm{ROI}}{\;-\;\mathrm{OD}}_{\mathrm{background}}}{{\mathrm{OD}}_{\mathrm{reference}}}\times 100.$$

Linear regression was performed to calculate expected values for the fibrocyte ablated group.

### 2.12. Eicosanoid Profiling

The concentrations of eicosanoids were assessed in pooled liver lysates (fibrocyte-ablated-, control-, and supercontrol-group, *n* ≥ 8 per group) via LC-MS/MS as described before [47].

### 2.13. Statistical Analysis

Statistical analysis was performed using GraphPad Prism, version 8.20 for Mac (GraphPad software, La Jolla, CA, USA). Unpaired t-test (two-tailed) was applied for hypothesis testing, if normal distribution of data was not negated after evaluating histograms and QQ-plots. If normal distribution of data was rejected, then the Mann-Whitney *U* test was applied. The significance level ⍺ was set to 0.05; *p*-values < 0.05 were labeled with an asterisk (*). For explorative analyses, unpaired t-tests (two-tailed) were performed where appropriate. No correction for multiple comparisons was applied. Data are depicted as means or median ± 95% confidence intervals or SEM.

## 3. Results

### 3.1. Suicide Gene Strategy Enabled the Depletion of Bone Marrow-Derived Fibrocytes

TAA- and VCV-treatment was well tolerated during the animal experiments. After reconstitution from bone marrow transplantation, mice gained weight steadily, regardless of group affiliation. Basic observational data are provided in Supplementary Figure S1. Due to drastic weight loss, one out of the 32 mice was euthanized ahead of schedule. A subsequently performed autopsy of the euthanized mouse remained inconclusive, yet confirmed the successful reconstitution of bone marrow.

RNA in situ hybridization was performed on liver sections to visualize the suicide gene strategy’s success. Bone marrow-derived fibrocytes were identified by the simultaneous expression of *Ptprc* (CD45) and *Col1a1* mRNA (collagen type I α-1 chain, Figure 1b). A considerable amount of fibrocytes, most frequently located in the periportal area and interportal septi, was detected in mice of the control group. While single cells remained, a marked reduction of the number of fibrocytes was observed in mice which received Col-HSV-TK bone marrow (Figure 1b, bottom).

### 3.2. Depletion of Fibrocytes Attenuated Hepatic Fibrogenesis

**Figure 2:**
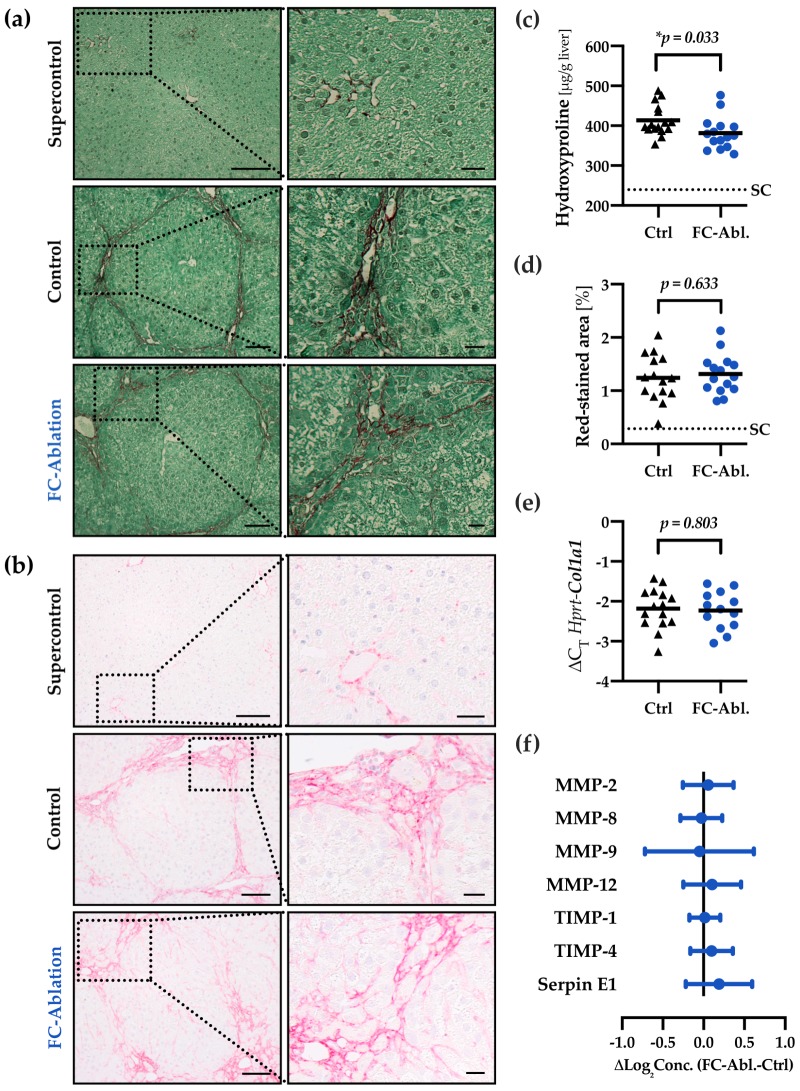
Fibrocyte ablation attenuated hepatic fibrogenesis. Fibrillar collagen distribution was visualized by (**a**) Sirius Red/Fast Green staining and (**b**) immunohistochemical staining of collagen I on formalin fixed, paraffin-embedded liver sections. TAA-treatment caused pronounced periportal and bridging fibrosis as well as faint chicken wire sinusoidal fibrosis in the control- and fibrocyte-ablated group. Dotted boxes are shown in enlarged panels on the right side. Magnification 200×, bars 100 and 25 µm. (**c**) Quantitative assessment of hepatic hydroxyproline content revealed a reduction of fibrillar collagens in mice lacking fibrocytes. The assay was performed three times. Mean values of each individual mouse are depicted by black triangles (control) or blue dots (fibrocyte ablation). The solid line depicts the group mean, the dotted line the mean hydroxyproline level in untreated supercontrols (SC). (**d**) Morphometric analysis of Sirius Red/Fast Green-stained sections displayed a comparable extent of red-stained areas in TAA-treated mice with and without fibrocyte ablation. A total of 1361 images were analyzed (2–134 per mouse). (**e**) The transcriptional levels of *Col1a1* were equal throughout both groups. (**f**) Relative protein levels of MMP-2, MMP-8, MMP-9, MMP-12, TIMP-1, TIMP-4, and Serpin E1 were assessed utilizing a multiplex ELISA and remained constant as a result of fibrocyte ablation. Absolute concentrations and individual *p*-values are provided in Figure S4.

TAA-administration induced a marked perilobular fibrosis in mice of the control (Ctrl)- and fibrocyte-ablated group (FC-Abl., Figure 2a,b). Irregular liver architecture and regenerative nodules indicated beginning cirrhosis in some mice. Total liver hydroxyproline content was reduced by 7.8% (95% CI: 0.7%–14.8%; Figure 2c) in fibrocyte-ablated mice, denoting a reduced deposition of fibrillar collagens. The difference is considered statistically significant (*p* = 0.033). Neither the pathologist’s staging depicted in Table 1, nor the morphometric analysis of histological samples (Figure 2d) showed differences between the control- and fibrocyte-ablated group.

Next, a high-throughput analysis of 84 fibrosis-related genes was conducted; a table of the strongest regulated genes can be found in Figure S3. The gene expression of *Col1a2* (fold-change 0.88) and *Col3a1* (fold-change 0.88) was not relevantly altered in this array. Quantitative real-time PCR, moreover, demonstrated an unchanged expression of *Col1a1* in result of the fibrocyte ablation (fold-change 0.97; 95% CI: 0.74–1.26; *p* = 0.803; Figure 2e).

The extent of fibrosis is largely influenced by the degradation of ECM components. Quantitative analysis of the protein levels of several MMPs and TIMPs showed an about equal expression throughout both groups (Figure 2f). The mean group difference of all but one analyte (MMP-9) yielded 95% confidence intervals whose border values were considered irrelevant in the context of this study. For MMP-9, the high scatter in the data, possibly due to the short half-life of MMP-9, impeded a conclusive interpretation. While there is a negligible mean difference, the 95% confidence interval ranges from a considerable increase (40.0%) to a noteworthy decrease (50.4%) in the mean concentration. Absolute concentrations and individual *p*-values are provided in Figure S4. The gene expression of selected MMPs and TIMPs was not relevantly altered in the gene expression array either.

### 3.3. The Antifibrotic Effect was Not Accompanied by A Reduction of Myofibroblasts

**Figure 3:**
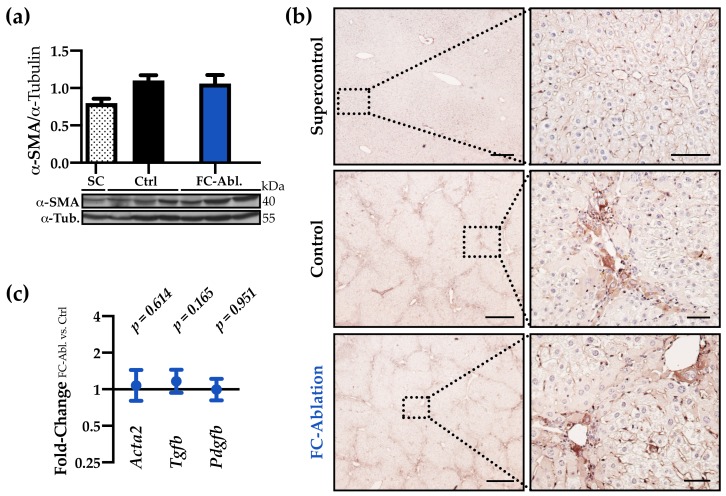
The antifibrotic effect was not accompanied by a reduction of myofibroblasts. (**a**) Western blot analysis and optical densitometry thereof revealed that the hepatic α-SMA levels were increased following TAA-treatment but unchanged by fibrocyte ablation. Two individual western blots were included in the analysis, a representative blot is shown. Arbitrary unit. *SC n* = 2; *Ctrl, FC-Abl. n* = 6. Mean + SEM is depicted. (**b**) Immunohistochemical staining of α-SMA (brown) demonstrated the periportal accumulation of myofibroblasts in TAA-treated animals and an unchanged expression pattern in result of the fibrocyte ablation. Representative stainings are shown. Magnification 40× and 200×, bars 400 and 50 µm. (**c**) Hepatic gene expression levels of *Acta2*, *Tgfb,* and *Pdgfb* were comparable at the end of the experiment (full data in Figure S5a–c).

Despite interindividual differences within the control- and fibrocyte-ablated group, the overall hepatic expression of α-SMA was comparable both on protein- (Figure 3a,b) and transcriptional level (fold-change 1.08; 95% CI: 0.80–1.44; *p* = 0.614; Figure 3c). Immunohistochemical staining (Figure 3b), furthermore, revealed a similar staining intensity and distribution pattern of α-SMA. Additionally, we investigated the gene expression of the most potent myofibroblast activators TGF-β and PDGF. Both were expressed about equally in the control and the fibrocyte-ablated group (fold-change 1.16 and 0.99; *p* = 0.165 and 0.951; Figure 3c, full data in Figure S5a–c).

### 3.4. Fibrocyte Ablation Lead to A Reduction of Hepatic IL-1β Levels

**Figure 4:**
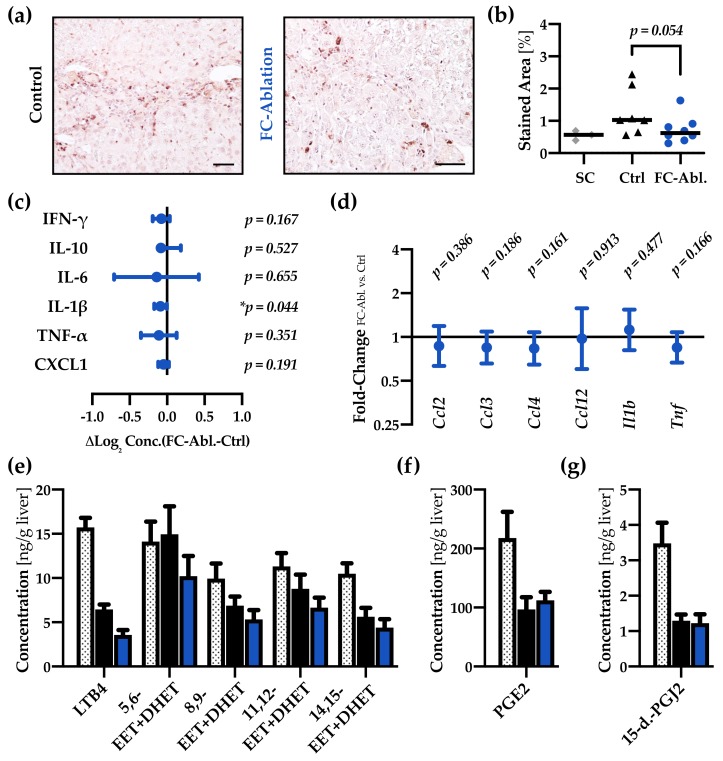
Fibrocyte ablation lead to a reduction of hepatic IL-1β levels. (**a**) Immunohistochemical staining of CD45 (grey) and (**b**) subsequent morphometric analysis revealed a tendentially reduced number of leukocytes in the liver of fibrocyte-ablated mice. Magnification 200×, bar 50µm. Mann-Whitney *U* test was applied. (**c**) Multiplex ELISA demonstrated a reduction of IL-1β protein levels while none of the other cytokines were significantly regulated. Absolute concentrations and individual *p*-values are provided in Figure S8. (**d**) qRT-PCR showed no regulations in a panel of inflammatory genes (full data in Figure S5d–i). (**e**–**g**) In comparison to healthy supercontrols (*n* = 8, dotted bars), absolute quantification of hepatic eicosanoids revealed a notable decrease of all but one analyte (5,6-EET + DHET) in consequence of the TAA-treatment. The level of LTB4 is considerably lower in FC-ablated mice (*n* = 15, blue), compared to controls (*n* = 15, black). Mean of three measurements + SEM are depicted.

Next, we evaluated the hepatic infiltration and proliferation of inflammatory cells. While histological grading, performed on routine hematoxylin/eosin-staining, did not retrieve significant differences in result of fibrocyte-ablation (Table S6), immunohistochemical staining and subsequent morphometric analysis of the pan-leucocyte marker CD45 hinted at a decreased number of CD45-positive cells in the liver (*p* = 0.054; Figure 4a,b).

In order to assess paracrine inflammatory functions of fibrocytes, we evaluated various cytokines on a transcriptional and protein level. Proteome Profiling of 111 cytokines was performed, yet none of the suspected mediators were strongly regulated (Figure S7). Multiplex ELISA-data of common inflammatory cytokines (Figure 4c, full data in Figure S8) revealed that the level of IL-1β was significantly decreased in fibrocyte-ablated mice (13.7 ± 0.98 vs. 12.9 ± 1.12 ng/g liver; *p* = 0.044). Moreover, genes encoding inflammatory markers (*Tnf*, *Ccl3*, and *Ccl12*) were among the strongest regulated analytes in the gene expression array (Figure S3). Significantly regulated- and further genes of interest were therefore assessed using quantitative real-time PCR (Figure 4d), yielding no significant regulations.

Since FC are known to express cysteinyl leukotrienes (CysLTs), we also sought to determine the hepatic levels of several eicosanoids (Figure 4e–g). While CysLTs were not detectable in our experimental setup, other changes in the eicosanoid profile occurred in response to the fibrogenic stimulus. The concentration of leukotriene B4 (LTB4), several isomers of epoxyeicosatrienoic acid (EETs), 15-deoxy-delta-12,14-prostaglandin J2 (15-d.-PGJ2), and prostaglandin E2 (PGE2) decreased in TAA-treated groups. FC ablation lead to a subtle attenuation of the EETs and a notable decrease of LTB4 (Figure 4e).

### 3.5. Liver Integrity was Ameliorated by Fibrocyte Depletion

**Figure 5:**
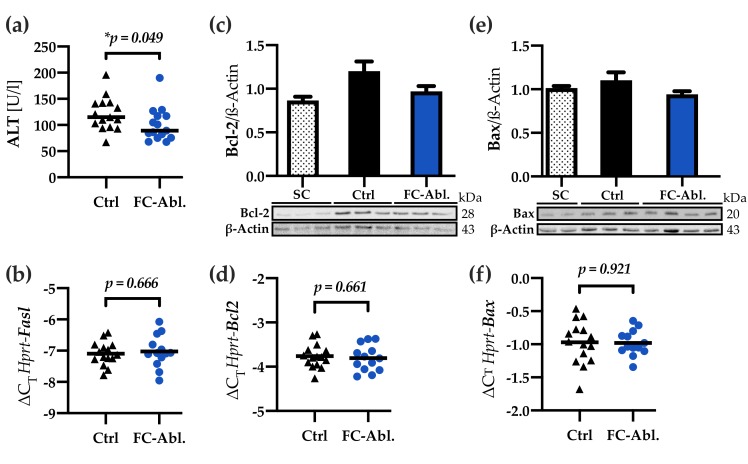
Liver integrity was preserved by fibrocyte ablation. (**a**) Fibrocyte ablation caused a considerable reduction in serum levels of alanine amino transferase (ALT). Median is depicted, Mann-Whitney *U* test was applied. (**b**) Quantitative real-time PCR showed an unchanged expression of *Fasl.* (**c**) Bcl-2 western blot and optical densitometry thereof hinted at a reduced expression of Bcl-2 in the fibrocyte-ablated group. Arbitrary unit. *SC n* = 5; *Ctrl, FC-Abl. n* = 6. (**d**) qPCR displayed an unchanged *Bcl2* expression. (**e**,**f**) Bax is expressed comparably on a protein and transcriptional level in the control- and fibrocyte-ablated group. Arbitrary unit; *SC n* = 4; *Ctrl n* = 10; *FC-Abl. n* = 9; All western blot experiments were performed ≥2 times, representative blots are shown. Columns and error bars depict Mean + SEM.

Cell death is a key event in the pathogenesis of liver fibrosis. Significantly reduced serum levels of alanine amino transferase (ALT) demonstrated a mitigation of liver-specific cellular damage in the fibrocyte-ablated group (−20.9%; 95% CI: 0.4–36.9%; *p* = 0.049; Figure 5a). High-throughput gene expression analysis indicated a decrease of the extracellular death ligand FAS (Figure S3), providing a possible explanation in the differential regulation of apoptosis. Subsequently performed quantitative real-time PCR, however, did not support a regulation of *Fasl* (fold-change 1.04; 95% CI: 0.83–1.34; *p* = 0.666; Figure 5b). Even more, further analyses displayed a subtle downregulation of the antiapoptotic factor Bcl-2 as a consequence of the depletion of fibrocytes (Figure 5c). Neither the transcriptional analysis of *Bcl2* (fold-change 0.97; 95% CI: 0.83–1.13; *p* = 0.661; Figure 5d), nor western blotting (Figure 5e) and quantitative real-time PCR of the Bcl-2 associated factor Bax (fold-change 0.99; 95% CI: 0.86–1.15; *p* = 0.921; Figure 5f) corroborated a general regulation regarding the Bcl-2 family.

### 3.6. A Four-Week Period is Not Sufficient to Provoke Regression of TAA-Induced Liver Fibrosis

**Figure 6:**
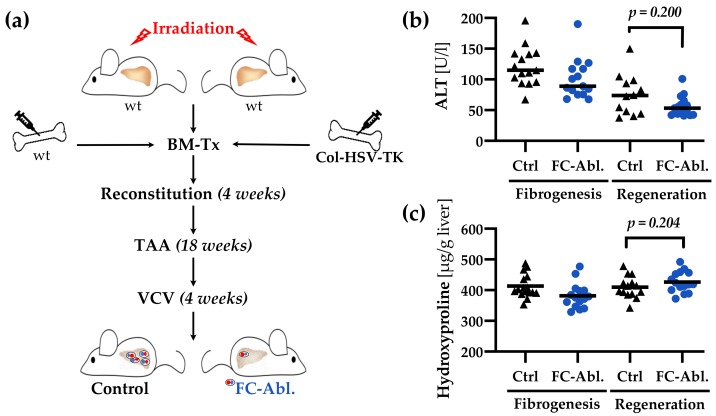
A four-week period was not sufficient to provoke regression of fibrosis. (**a**) Schematic representation of the animal experiments. Fibrocytes were depleted in mice of the respective group via the administration of VCV during a four-week regeneration period. (**b**) The direct comparison of serum ALT levels of mice sacrificed during fibrogenesis (see Section 3.5) and after regeneration shows an ameliorated disease state four weeks after the last TAA-administration. FC depletion during regeneration did not alter ALT levels. Median is depicted, Mann-Whitney *U* test was applied. (**c**) The direct comparison of hepatic hydroxyproline content indicates that a resolution of fibrosis cannot be observed after a regeneration period as short as four weeks. FC depletion during regeneration did not influence hydroxyproline levels. Mean is depicted, an unpaired *t*-test (two-tailed) was applied.

Lastly, we aimed to study the effects of a fibrocyte depletion on hepatic fibrolysis and regeneration. FC were depleted during a 4-week regeneration period after 18 weeks of fibrosis induction (Figure 6a). As the TAA-model induced a favorable, marked fibrosis, representing typical pathological features known from human liver fibrosis as expected (Figure 2), we anticipated a considerable regeneration after 4 weeks. Serum ALT levels, in fact, suggest an ameliorated disease state (Figure 4b). Surprisingly, fibrosis was not reduced by a 4-week regeneration period after cessation of the noxe: Total liver hydroxyproline levels remained constant in the control group after the regeneration period (409.8 ± 35.5 vs. 413.4 ± 38.2 µg/g liver; Figure 6c). FC depletion during the regeneration period did not significantly alter either hydroxyproline content (Figure 6c) or serum ALT levels (Figure 6b). We chose to not further investigate the role of FC in fibrolysis and regeneration until a protocol leading to marked liver regeneration could be established for the TAA-model.

## 4. Discussion

In light of recent translational approaches in the field of liver fibrosis, a thorough understanding of fibrocyte biology is urgently needed. Bone marrow transplantation, for instance, proved beneficial in murine cholestatic fibrosis but remains controversial as a treatment option for chronic liver diseases [48]; Cenicriviroc (CVC), a dual CCR2/CCR5-inhibitor impeding the infiltration of monocytes, is a promising drug candidate for NAFLD patients with fibrosis [49,50]. Fibrocytes potentially contribute to the targeted monocyte population and are known to be recruited via CCR2, -3, -5, and -7 signaling [8,20,23,51,52]. CVC just recently provided promising results reducing fibrosis but surprisingly not reducing inflammation in NAFLD-patients, and is currently tested in a phase III trial (NCT03028740) [53].

We herein present a novel approach to investigate the role of bone marrow-derived fibrocytes in liver fibrosis. Our model enabled the specific depletion of fibrocytes, avoiding the dependence on particular surface markers or differentiation pathways. RNA in situ hybridization, however, implies that it did not entirely deplete bone marrow-derived fibrocytes (Figure 1b). This result appears in line with evidence from a study Puche et al. conducted: utilizing the HSV-TK model, about 65% of HSCs could be depleted in a CCl_4_-model of hepatic fibrosis [54]. Even though our analyses suggest a superior depletion-rate, it should be a concern of future studies to closely monitor the depletion effectiveness to not underestimate the role of bone marrow-derived fibrocytes.

Our results show a functional contribution of fibrocytes to hepatic fibrogenesis. The determination of hydroxyproline content revealed a reduced deposition of fibrillar collagens as a result of the depletion of fibrocytes (Figure 2c). The present data allow a range of interpretations regarding the extent of the mitigation: While the common prediction, mainly based on fate-tracing studies and evidence from other organs, that the depletion of fibrocytes yields minor effects on hepatic fibrogenesis [15,17,55] is compatible with our data, the 95% confidence interval also spans a reduction of up to ∼15%. An attenuation of that magnitude is considered highly clinically significant and would challenge our understanding of the contribution of fibrocytes. Significantly reduced serum ALT levels (Figure 5a), despite the generally moderate level of hepatocyte damage, support this notion. It appears noteworthy that (1) the reduction of hydroxyproline was not accompanied by a changed gene expression of collagen I in our study and (2) contradicting results were obtained in studies, investigating the contribution of fibrocytes to fibrosis of the liver [39] and lung [56].

The unchanged gene expression of *Col1a1*, *Col1a2,* and *Col3a1* (Figure 2e and Figure S3) implies that a reduced secretion of collagens at the time of analysis is not the cause of the reduced hydroxyproline content. The development of fibrosis is highly dependent upon the balance of deposition and degradation of ECM-material [57]. Although fibrocytes are known to express several MMPs [30,31], the overall hepatic expression of these was unchanged in result of the fibrocyte ablation (Figure 2f), too. We therefore hypothesize that the mitigation of fibrosis is the result of a transient regulation of fibrogenesis during the disease progression. Given their properties as hematopoietic, circulating cells, fibrocytes can be found early at the site of injury [3,20]. Investigations regarding the influx of fibrocytes into the injured liver show a peak two weeks after onset of the fibrogenic stimulus [20]. It has to be considered, however, that fibrosis was induced via CCl_4_ in this experiment, which provokes an accelerated disease progression, compared to the TAA model [41,42]. Taken together, these results suggest that our timepoint of analysis missed the greatest contribution of fibrocytes yet displayed a lasting effect and provide encouragement to more closely focus on the role of fibrocytes in different stages of disease progression in future research.

Ozono et al. just recently published their findings as a clodronate liposome-mediated depletion of fibrocytes »had little contribution on liver fibrosis« in a murine model of CCl_4_-induced fibrosis [39]. Although the authors concluded differently, we argue that the results they present are not necessarily contradictory to ours. Morphometric analysis displays a reduction of stained fibrillar collagens by tendency (see Figure 3b in reference [39]). Smaller sample sizes (*n* = 8) might provide an explanation of why the level of statistical significance postulated by the authors was not reached. Furthermore, semiquantitative means like histology with subsequent pathological evaluation (staging) or morphometric analysis perhaps lack the accuracy to detect subtle changes in the deposition of ECM components. Consistent with this claim, semiquantitative methods failed to detect the mitigation of fibrosis in our study (Table 1, Figure 2d). Solely relying on those techniques might under some circumstances therefore be inadequate to elaborate the biology of fibrocytes. Even though it will be inevitable to study the contribution of fibrocytes in different models of fibrosis, the use of distinct models and readout parameters impedes the comparability of results obtained with such.

A specific knockout of the *Col1a1* gene in fibrocytes, furthermore, yielded no significant impact on pulmonary fibrosis, even though up to 30% of collagen producing cells are assumed to be fibrocytes, suggesting a crucial role of paracrine functions [56]. We herein sought to investigate effects on the activation and proliferation of myofibroblasts, hepatic inflammation, and cell death. Since there is compelling evidence for activated HSCs being the main contributors to hepatic collagen deposition [15,16,17], and fibrocytes, in fact, can facilitate the activation of myofibroblast via the secretion of TGF-β and PDGF in vitro [26], a decreased activation of myofibroblasts might provide a plausible explanation for the observed attenuation of fibrosis. Even though a transient process cannot be excluded, our results, showing an unchanged expression of α-SMA, *Tgfb,* and *Pdgfb* in result of the fibrocyte depletion (Figure 3), tend to refute this hypothesis. The reduced CD45-stained area (Figure 4a,b) and the decreased hepatic concentration of IL-1β (Figure 4c) might imply an ameliorated inflammatory response in consequence of the fibrocyte depletion. Nevertheless, the evaluation of inflammatory cytokines (Figure 4c,d, Figure S7) emphasized that bone marrow-derived fibrocytes are not a major source of inflammatory mediators at the time of analysis. These results are noteworthy, given the fact that previous research provided evidence for a participation of fibrocytes in inflammatory processes [26,34,58] and entities like scleroderma, rheumatoid diseases, and asthma are associated with fibrocytes (reviewed in reference [24]). Our findings call for careful considerations, especially regarding the interpretation of cultivation and stimulation experiments performed with fibrocytes. Lastly, the decreased levels of serum ALT (Figure 5a) can be interpreted as a result of ameliorated fibrosis. They might, however, also display an attenuation of hepatic cell death, caused by the depletion of fibrocytes, leading to reduced profibrogenic stimuli. Serum amyloid P, which is known to inhibit the differentiation of fibrocytes [59], prevented hepatic cell damage in CCl_4_-induced acute liver injury [60]. While multiple forms of hepatic cell death are known [61], we found subtle regulations regarding apoptosis. Contradictory and partly not reproducible results herein forbid a conclusive interpretation.

In summary, we herein demonstrate a functional contribution of bone marrow-derived fibrocytes to hepatic fibrogenesis. However, a definitive mode of action could not be identified. It has to be considered that neither our analyses of paracrine fibrocyte functions, despite covering the crucial mediators of hepatic fibrogenesis, were exhaustive nor the previous cultivation and fate-tracing studies necessarily elucidated the entire range of fibrocyte functions in a complex in vivo setting. Since it is, due to the high plasticity and little number of cells, often troublesome to study fibrocytes in vivo, it is noteworthy that properly planned animal experiments with a rigorous statistically substantiated design according to the 3R principles enabled the generation of robust results. Fibrocytes should be considered in future research to acquire a thorough understanding of the biology of hepatic fibrosis.

## Figures and Tables

**Table 1:** Staging according to Ishak et al.

Stage	0	1	2	3	4	5	6	Median
Supercontrol	3	5	0	0	0	0	0	**1**
Control	0	0	0	8	6	2	0	3.5 ^1^
FC-Ablation	0	0	0	6	5	4	0	4 ^1^

^1^*p* = 0.476; Mann-Whitney *U* test was applied.

## Supplementary Materials

The following are available online at https://www.mdpi.com/2073-4409/8/10/1210/s1, Figure S1: Schematic gene construction and basic experimental data, Table S2: Primer sequences used in quantitative real-time PCR, Figure S3: Gene Expression Array results, Figure S4: Hepatic protein concentrations of MMPs and TIMPs, Figure S5: Detailed quantitative real-time PCR results, Table S6: Grading according to Ishak et al., Figure S7: Proteome Profiling of inflammatory cytokines, Figure S8: Hepatic protein concentrations of inflammatory cytokines.
