# Supplementary material for: Depletion of Bone Marrow-Derived Fibrocytes Attenuates TAA-Induced Liver Fibrosis in Mice
Source: Cells. 2019 Oct 7;8(10):1210. doi: 10.3390/cells8101210 (PMC6829877; doi:10.3390/cells8101210)
Supplement: Supplementary file 1 [file cells-08-01210-s001.pdf]

cells

*Supplementary material*

# Depletion of Bone Marrow-Derived Fibrocytes Attenuates TAA-Induced Liver Fibrosis in Mice

**Felix Hempel [1,†], Martin Roderfeld [1,†], Rajkumar Savai [2,3], Akylbek Sydykov [3], Karuna Irungbam [1], Ralph Schermuly [3], Robert Voswinckel [4,5], Kernt Köhler [6], Yury Churin [1], Ladislau Kiss [3], Jens Bier [3], Jörn Pons-Kühnemann [7] and Elke Roeb [1,*]**

1 Department of Gastroenterology, Justus Liebig University, D-35392 Giessen, Germany; felix.hempel@med.jlug.de (F.H.) (https://orcid.org/0000-0002-2733-4313); martin.roderfeld@innere.med.uni-giessen.de (M.R.); kukuirungbam@gmail.com (K.I.); yury.churin@innere.med.uni-giessen.de (Y.C.)
2 Max Planck Institute for Heart and Lung Research, Member of the German Center for Lung Research (DZL), Member of the Cardio-Pulmonary Institute (CPI), D-61231 Bad Nauheim, Germany; rajkumar.savai@mpi-bn.mpg.de (https://orcid.org/0000-0003-1538-2091)
3 Department of Internal Medicine, Cardio-Pulmonary Institute (CPI), Universities of Giessen and Marburg Lung Center (UGMLC), Member of the German Center for Lung Research (DZL), Justus Liebig University, D-35392 Giessen, Germany; akylbek.sydykov@innere.med.uni-giessen.de (A.S.) (https://orcid.org/0000-0002-8122-7033); ralph.schermuly@innere.med.uni-giessen.de (R.S.) (https://orcid.org/0000-0002-5167-6970); ladislau.kiss@innere.med.uni-giessen.de (L.K.); jens.bier@innere.med.uni-giessen.de (J.B.)
4 Department of Internal Medicine, Bürgerhospital, D-61169 Friedberg, Germany; robert.voswinckel@gz-wetterau.de
5 Department of Internal Medicine, Hochwaldkrankenhaus, D-61231 Bad Nauheim, Germany
6 Institute of Veterinary Pathology, Justus Liebig University, D-35392 Giessen, Germany; kernt.koehler@vetmed.uni-giessen.de
7 Institute of Medical Informatics, Justus Liebig University, D-35392 Giessen, Germany; joern.pons@informatik.med.uni-giessen.de (https://orcid.org/0000-0002-8211-4399)
† These authors contributed equally
* Correspondence: elke.roeb@innere.med.uni-giessen.de; Tel.: +49-641-985-42338

**Content:**

Supplementary Figure S1: Schematic plasmid construction and basic experimental data
Supplementary Table S2: Primer sequences used in quantitative real-time PCR
Supplementary Figure S3: Gene Expression Array results
Supplementary Figure S4: Hepatic protein concentrations of MMPs and TIMPs
Supplementary Figure S5: Detailed quantitative real-time PCR results
Supplementary Table S6: Grading according to Ishak *et al.*
Supplementary Figure S7: Proteome Profiling of inflammatory cytokines
Supplementary Figure S8: Hepatic protein concentrations of inflammatory cytokines

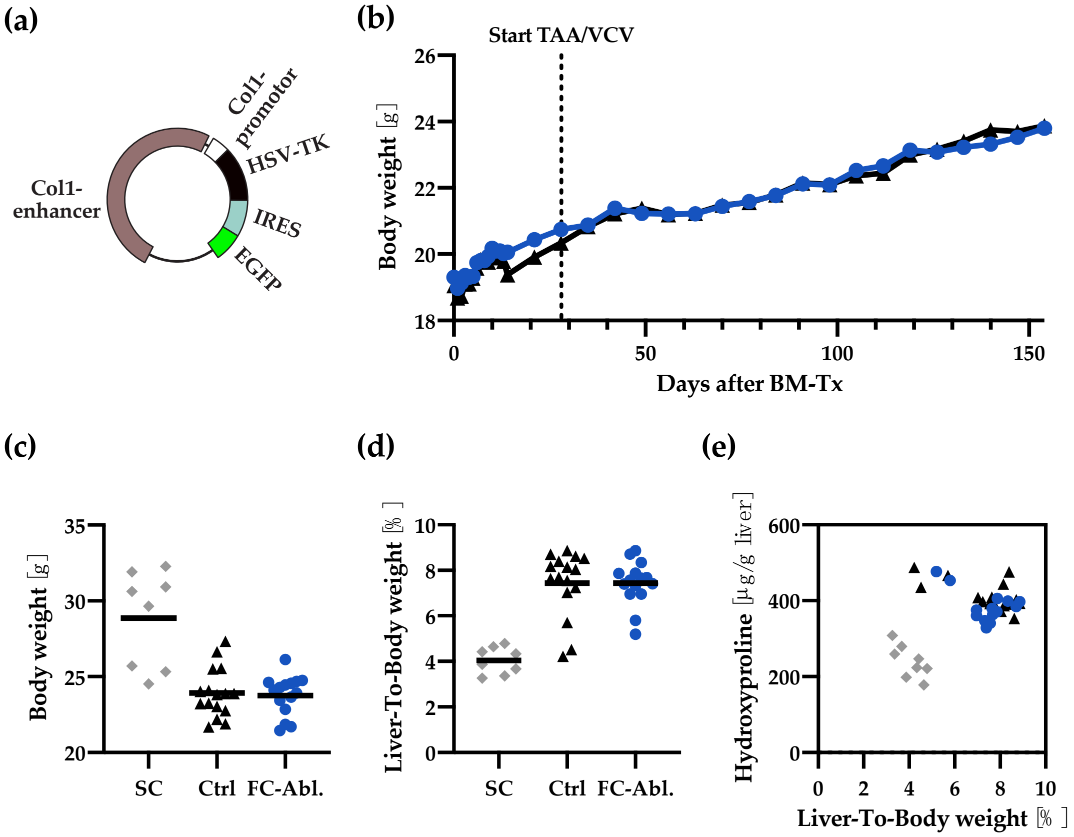


**Supplementary Figure S1: Schematic plasmid construction and basic experimental data.** (**a**) Schematic representation of the fusion gene construct used for the development of transgenic mice expressing HSV-TK from the type I collagen promoter [38]. Transgenic mice served as bone marrow donors for mice of the fibrocyte ablation group. **(b)** Body weight development of the control (n=16, black triangles) and fibrocyte-ablated group (n=15, blue dots). There was no statistically significant difference at any timepoint, indicated by a fitted mixed-effect model. **(c)** Final body weight. Grey diamonds depict untreated supercontrols (n=8). **(d)** Liver-to-body weight ratios. **(e)** Hydroxyproline levels plotted against liver-to-body weight ratios. The scatter plot shows that all mice, regardless of the wide scatter in liver-to-body weight, developed fibrosis, indicated by increased hepatic hydroxyproline content.

| Gene | Primer | Sequence (5′-3′) |
|---|---|---|
| ***Hprt*** | forward | GGCCTCCCATCTCCTTCATG |
| | reverse | CAGTCCCAGCGTCGTGATTA |
| ***Col1a1*** | forward | GCTCCTCTTAGGGGCCACT |
| | reverse | CCACGTCTCACCATTGGGG |
| ***Ccl2*** | forward | TTAAAAACCTGGATCGGAACCAA |
| | reverse | GCATTAGCTTCAGATTTACGGGT |
| ***Ccl3*** | forward | TTCTCTGTACCATGACACTCTGC |
| | reverse | CGTGGAATCTTCCGGCTGTAG |
| ***Ccl4*** | forward | TTCCTGCTGTTTCTCTTACACCT |
| | reverse | CTGTCTGCCTCTTTTGGTCAG |
| ***Ccl12*** | forward | ATTTCCACACTTCTATGCCTCCT |
| | reverse | ATCCAGTATGGTCCTGAA |
| ***Il1b*** | forward | CCAGCTTCAAATCTCACAGCAG |
| | reverse | CTTCTTTGGGTATTGCTTGGGATC |
| ***Tnf*** | forward | CCCTCACACTCAGATCATCTT |
| | reverse | GCTACGACGTGGGCTACAG |
| ***Fasl*** | forward | TCCGTGAGTTCACCAACCAAA |
| | reverse | GGGGGTTCCCTGTTAAATGGG |
| ***Bcl2*** | forward | CTTCGCAGAGATGTCCAGTC |
| | reverse | CATCTCCCTGTTGACGCTC |
| ***Bax*** | forward | GGCAACTTCAACTGGGG |
| | reverse | CCACCCTGGTCTTGGATC |

**Supplementary Table S2: Primer sequences used in quantitative real-time PCR.** QuantiTect Primer Assays (QIAGEN, Hilden, Germany) were used to detect *Acta2*, *Tgfb*, and *Pdgfb*.

**(a)**

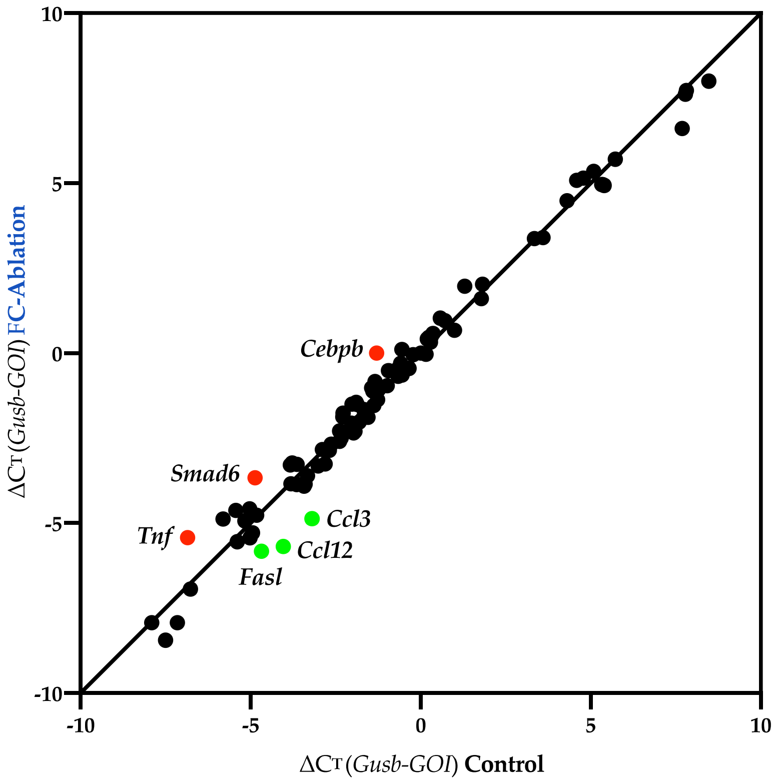


**(b)**

| Gene | Fold-Regulation (FC-Abl. vs. Ctrl) |
|---|---|
| *Cebpb* | 2.45 |
| *Smad6* | 2.3 |
| *Tnf* | $2.7^{\dagger}$ |
| *Ccl12* | $-2.23^{*}$ |
| *Ccl3* | -3.19 |
| *Fasl* | $-3.12^{*}$ |

**Supplementary Figure S3: Gene Expression Array results. (a)** Pooled samples of the control- and fibrocyte-ablated group (n=15 per group) were analyzed. The scatter plot depicts $\Delta C_T$ values of each of the 84 genes of interest (GOI), normalized to *Gusb*. Genes up- (red) or downregulated (green) more than two-fold are labeled. **(b)** Individual regulations of the six most regulated genes. Fold-regulations were obtained using the QIAGEN data analysis web portal. **one or †both of the $C_T$ values were >30 but <35.*

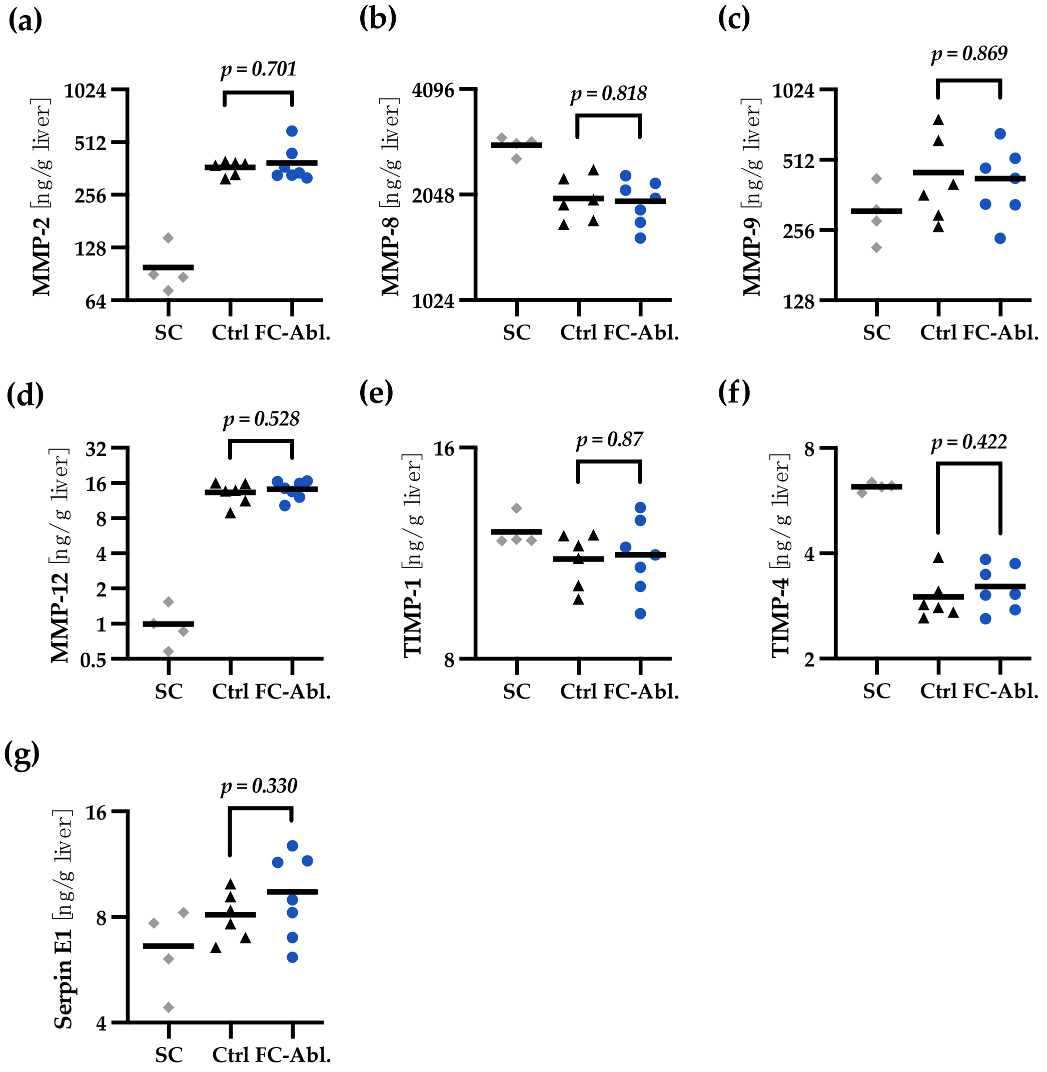


**Supplementary Figure S4: Hepatic protein concentrations of MMPs and TIMPs**. Concentration of **(a)** MMP-2, **(b)** MMP-8, **(c)** MMP-9, **(d)** MMP-12, **(e)** TIMP-1, **(f)** TIMP-4, and **(g)** Serpin E1/PAI-1. Measurements were performed using Magnetic Luminex Assays. *p*-values were calculated using unpaired t-test (two-tailed).

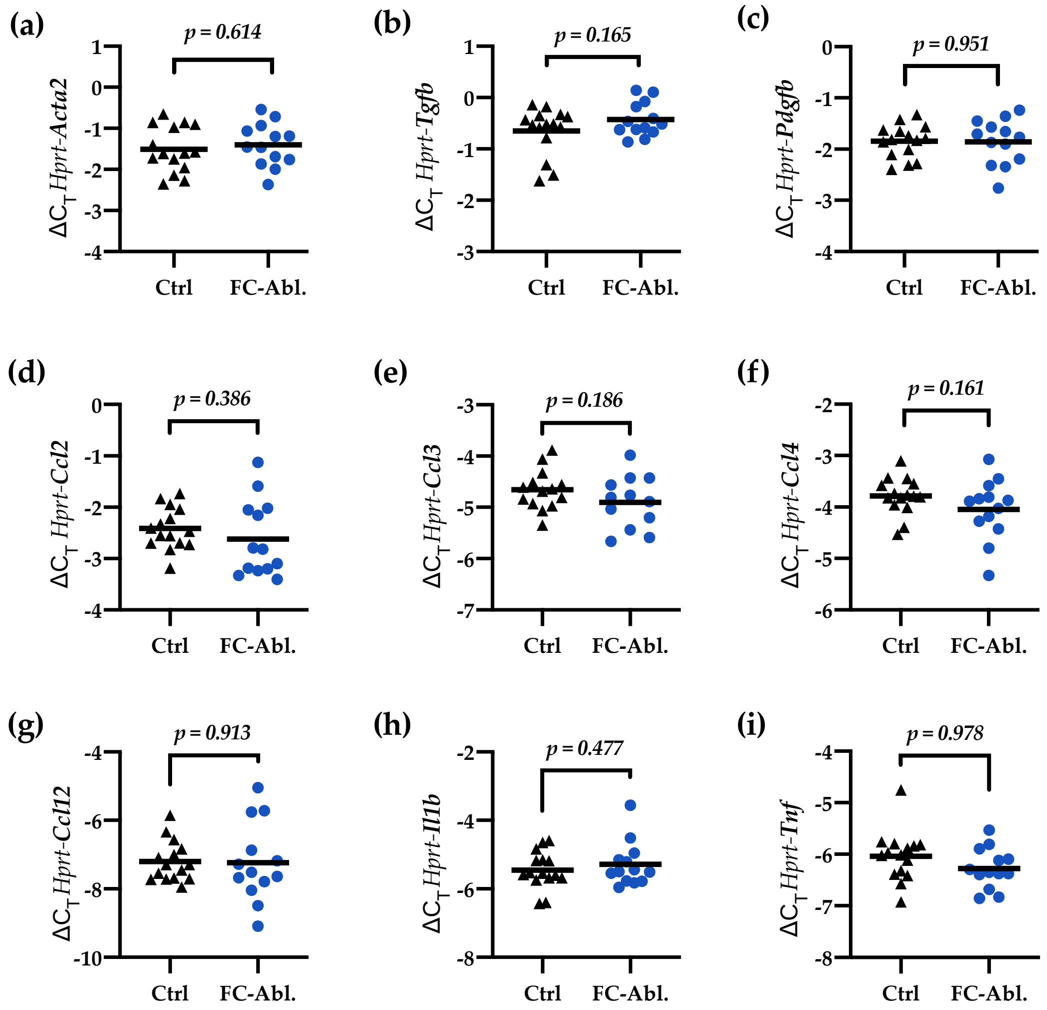


**Supplementary Figure S5: Detailed quantitative real-time PCR results.** Individual $\Delta C_T$ values of **(a)** *Acta2*, **(b)** *Tgfb*, **(c)** *Pdgfb*, **(d)** *Ccl2*, **(e)** *Ccl3*, **(f)** *Ccl4*, **(g)** *Ccl12*, **(h)** *Il1b*, and **(i)** *Tnfa*, each normalized to *Hprt*. Measurements were performed twice. *p*-values were calculated using unpaired t-test (two-tailed).

| Category | Group | **0** | **1** | **2** | **3** | **4** | **5** | **6** | **Med.** |
|---|---|---|---|---|---|---|---|---|---|
| A | **Supercontrol** | **6** | **2** | 0 | 0 | 0 | | | **0** |
| | **Control** | 0 | 0 | **1** | **11** | **4** | | | **3** |
| | **FC-Ablation** | 0 | 0 | 0 | **8** | **7** | | | **3** |
| B | **Supercontrol** | **8** | 0 | 0 | 0 | 0 | 0 | 0 | **0** |
| | **Control** | **16** | 0 | 0 | 0 | 0 | 0 | 0 | **0** |
| | **FC-Ablation** | **15** | 0 | 0 | 0 | 0 | 0 | 0 | **0** |
| C | **Supercontrol** | **6** | **2** | 0 | 0 | 0 | | | **0** |
| | **Control** | **3** | **13** | 0 | 0 | 0 | | | **1** |
| | **FC-Ablation** | **1** | **14** | 0 | 0 | 0 | | | **1** |
| D | **Supercontrol** | **6** | **2** | 0 | 0 | 0 | | | **0** |
| | **Control** | **8** | **8** | 0 | 0 | 0 | | | **1.5** |
| | **FC-Ablation** | **10** | **5** | 0 | 0 | 0 | | | **1** |

**Supplementary Table S6: Grading according to Ishak *et al.*** **(A)** Periportal or periseptal interface hepatitis (piecemal necrosis), **(B)** confluent necrosis, **(C)** focal (spotty) lytic necrosis, apoptosis and focal inflammation and **(D)** portal inflammation were rated [44]. Grading was performed by an experienced pathologist, evaluating routine hematoxylin/eosin-stained sections in a blinded fashion. Number of sections assigned to each grade and median grade of the group are given. No significant differences between control- and fibrocyte-ablated group were observed using Mann-Whitney *U* test.

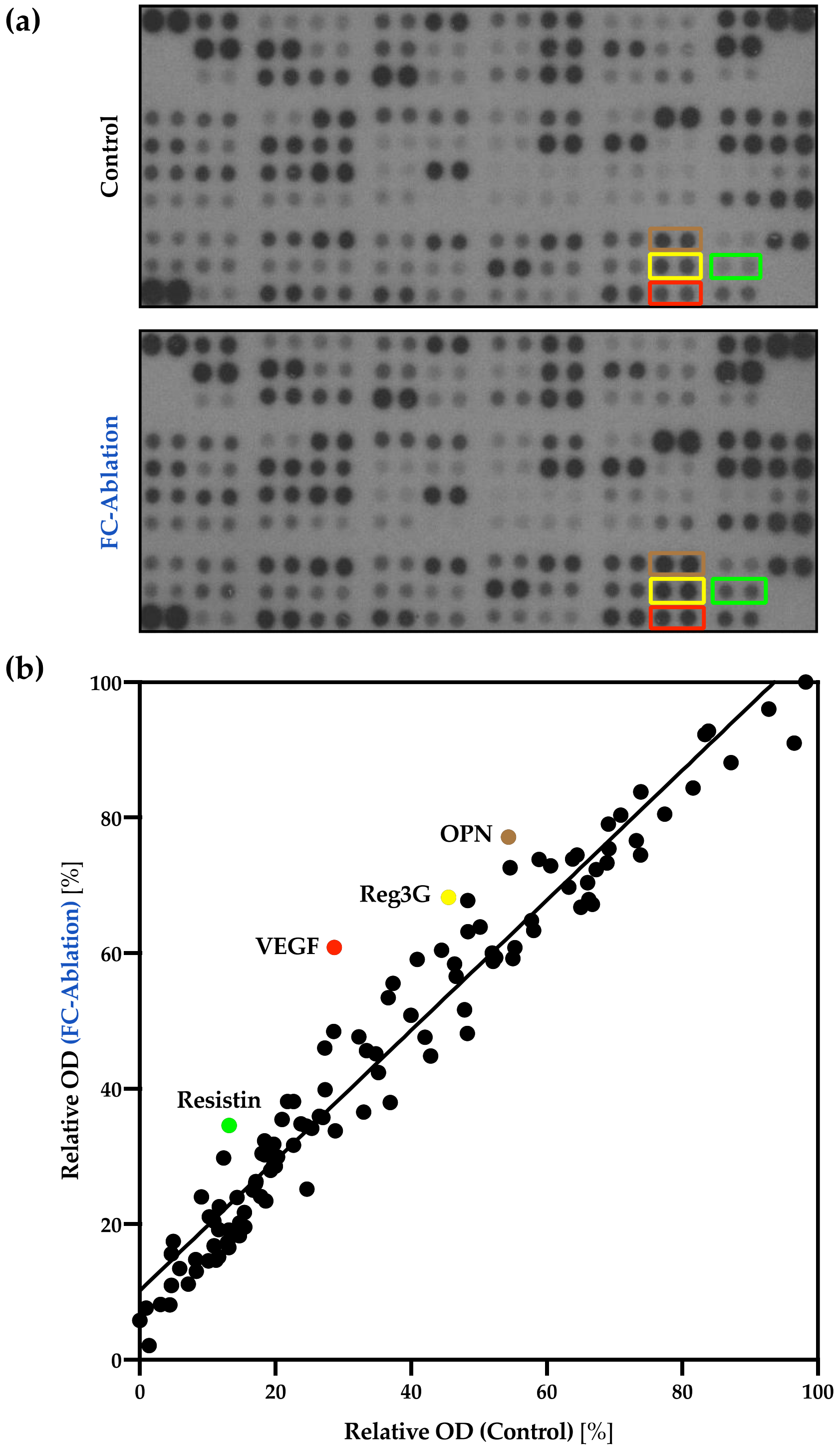


**Supplementary Figure S7: Proteome Profiling of inflammatory cytokines. (a)** High resolution scans of original arrays. **(b)** After background subtraction, each pair of dots was assigned a relative OD-value from 0%-100%, relative to the pair of dots with the highest OD. Relative ODs of the control and fibrocyte-ablated group are plotted. Linear regression was performed on the whole dataset (y=0.96x+10.15), deviations from the regression curve were interpreted as regulations. The corresponding dots of the most differentially expressed proteins are labeled in **(a)**.

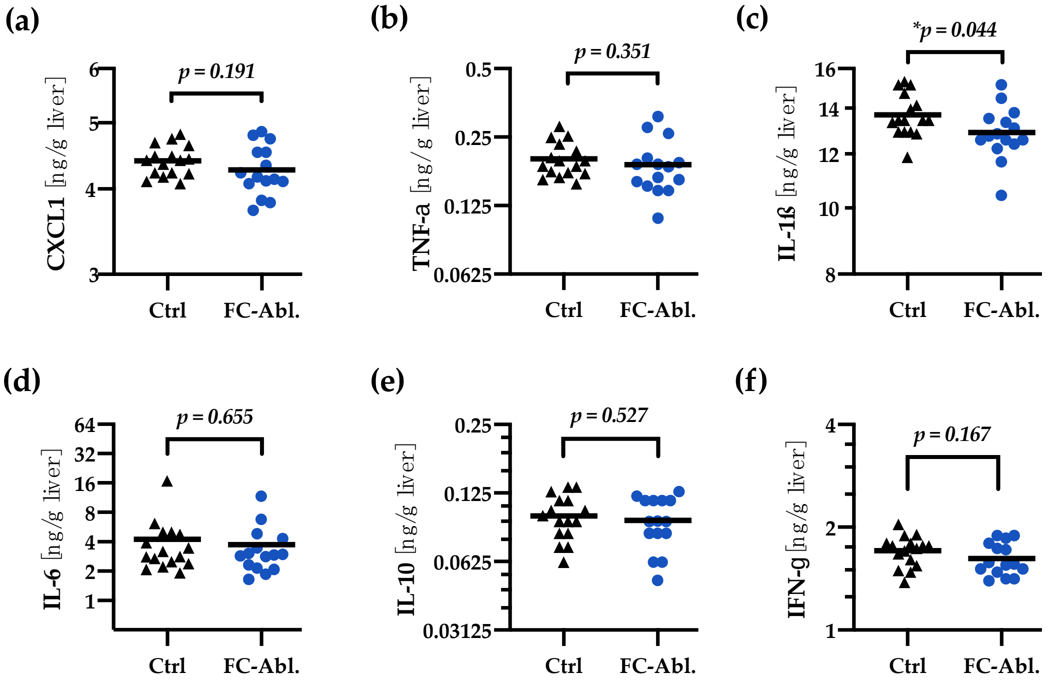


**Supplementary Figure S8: Hepatic protein concentrations of inflammatory cytokines.** Concentration of **(a)** CXCL1, **(b)** TNF-$\alpha$, **(c)** IL-1ß, **(d)** IL-6, **(e)** IL-10 and **(f)** IFN-$\gamma$. Measurement was performed using Multiplex ELISAs. *p*-values were calculated using unpaired t-tests (two-tailed).
